# Is retinal vein occlusion highly associated with an increased risk of myocardial infarction? A systematic review and meta-analysis

**DOI:** 10.1186/s40942-024-00606-9

**Published:** 2024-11-12

**Authors:** Kai-Yang Chen, Hoi-Chun Chan, Chi-Ming Chan

**Affiliations:** 1 School of Medicine, College of Medicine, Taipei Medical University, Taipei, Taiwan; 2 School of Pharmacy, China Medical University, Taichung, Taiwan; 3 Department of Ophthalmology, Cardinal Tien Hospital, New Taipei City, Taiwan; 4 School of Medicine, Fu Jen Catholic University, New Taipei City, Taiwan

**Keywords:** Retinal vein occlusion, Myocardial infarction, Cardiovascular risk, Central retinal vein occlusion, Branch retinal vein occlusion, Systematic review, Meta-analysis

## Abstract

**Background and objective:**

Retinal vein occlusion (RVO) and acute myocardial infarction (MI) are significant vascular events that impact patient health and mortality. Both conditions share common risk factors, such as hypertension, diabetes, and atherosclerosis. This study investigated the potential connection between RVO and MI, particularly among younger individuals, to improve preventive measures and management protocols.

**Method:**

A systematic review and meta-analysis were conducted, adhering to the PRISMA and MOOSE guidelines. Multiple databases, including PubMed, Scopus, MEDLINE, ScienceDirect, and ClinicalTrials.gov, were exhaustively searched until August 24, 2024. Studies were selected based on their reports of the association between RVO and MI risk. Quality assessment was performed using the Newcastle-Ottawa Quality Assessment Scale, and data were pooled using a random-effects model with hazard ratios and 95% confidence intervals.

**Result:**

Twelve studies comprising 371,817 participants were included. Meta-analysis revealed a pooled hazard ratio of 1.324 (95% CI, 1.238–1.415), indicating a significant association between RVO and increased MI risk (*p* = 0.0001). Subgroup analysis for central retinal vein occlusion (CRVO) showed a hazard ratio of 1.691 (95% confidence interval [CI] 1.142, 2.502, *p* = 0.009) with moderate heterogeneity (I^2^ = 36%), whereas branch retinal vein occlusion (BRVO) yielded a non-significant hazard ratio of 1.167 (95% CI 0.843, 2.106, *p* = 0.444; I^2^ = 33%). Publication bias was identified (Egger’s test, *p* = 0.036) and addressed through trim-and-fill adjustment, maintaining statistical significance.

**Conclusion:**

Our meta-analysis shows a strong association between CRVO and a 69.1% increased risk of MI, while BRVO shows no significant correlation. Overall, RVO is linked to a 32.4% elevated risk of MI. Despite slight publication bias, adjusted analyses confirm reliability, indicating that improved cardiovascular monitoring for RVO patients, especially those with CRVO, is essential to mitigate MI risk.

**Clinical trial number:**

Not applicable.

**Supplementary Information:**

The online version contains supplementary material available at 10.1186/s40942-024-00606-9.

## Introduction

Retinal vein occlusion (RVO) and myocardial infarction (MI) are two significant vascular events that significantly affect patient health and mortality [1, 2]. RVO occurs when the vein that supplies blood to the retina is blocked, resulting in sudden vision loss and indicating underlying systemic vascular disorders [3, 4]. MI, commonly known as a “heart attack,” occurs when coronary arterial blood flow to a portion of the heart is obstructed, causing damage to the heart muscle. This event can lead to hemodynamic deterioration and sudden death. The majority of MI result from underlying coronary artery disease, which remains the primary cause of mortality in the United States. When the coronary artery is occluded, the myocardium is deprived of oxygen [5, 6]. Both conditions share common risk factors such as hypertension, diabetes, and atherosclerosis, and are major contributors to cardiovascular-related mortality [7, 8].

The effects of these conditions on mortality were significant. Patients diagnosed with RVO face an increased risk of cardiovascular events, including stroke and MI [9–13]. This association underscores the importance of conducting comprehensive cardiovascular evaluations and providing appropriate management to individuals with RVO. Although medical advancements have led to improvements in the treatment of MI, it remains a major cause of death globally. In a systematic review and meta-analysis, the global prevalence of MI was found to be 3.8% in people aged < 60 years (*n* = 29.826.717) and 9.5% in people aged > 60 years (*n* = 5,071,185) [14, 15]. Although the overall morbidity and mortality rates for MI have decreased, hospitalization rates for young patients have not followed a similar downward trend [16]. This trend is particularly concerning in countries such as Korea, where MI rates among the young have risen, while declining among the elderly population [16].

This study aimed to investigate the possible association between RVO and MI, especially among younger individuals. Recognizing the shared risk factors and their significant influence on mortality, understanding this relationship could lead to improved preventive measures and management protocols for RVO patients. This meta-analysis aimed to evaluate the association between RVO and MI statistically, thereby addressing a critical gap in the current literature and providing a basis for future research and clinical guidelines.

## Methods

### Search strategy

We conducted an exhaustive investigation of multiple databases, including PubMed/Medline, Scopus, Science Direct, and Clinicaltrials.gov, adhering to the Preferred Reporting Items for Systematic reviews and Meta-Analyses (PRISMA) and Meta-Analysis of Observational Studies in Epidemiology (MOOSE) guidelines [17, 18]. A comprehensive search was performed using all available data from August 24, 2024. The search strategy utilized a range of MeSH terms, such as “retinal vein occlusion,” “myocardial infarction,” “risk factors,” “epidemiology,” and “cardiovascular diseases,” cardiovascular diseases. Boolean operators (AND, OR, and NOT) were employed to construct the search strategies and refine the search results. To ensure that no important publications were overlooked, a snowballing approach was used, and manual searches of the reference lists of eligible articles were performed to avoid missing relevant sources. The search strategy was developed independently by two authors (K. Y. C. and H.C.C.) according to the specified criteria, and any discrepancies or misunderstandings were resolved through consensus with a third author (C. M. C.). Our systematic review has been registered on an online registration website, PROSPERO, the number is CRD42024557823.

### Study selection

Eligible studies were identified based on the following criteria: original research articles reporting the association between RVO and risk of MI, with effect estimates in the form of risk, hazard, or odds ratios. A manual search included gray literature, such as conferences, posters, or bibliographies. Additional studies were identified by reviewing references of relevant papers. Titles and abstracts were screened to identify potentially eligible studies, duplicates were removed, and full-text articles were reviewed to confirm eligibility based on predefined inclusion criteria. No language restrictions were applied. Review articles, case series, editorials, letters, and other nonoriginal research articles were excluded. Studies needed sufficient methodological details and statistical analyses for a comprehensive assessment and synthesis.

### Eligibility criteria

The PECO framework (Population, Exposure, Comparison, Outcome) was used to establish the inclusion and exclusion criteria for this meta-analysis.

- **Population**: Adults diagnosed with RVO.
- **Exposure**: Observation of individuals with RVO.
- **Comparison**: Individuals without RVO or with other retinal conditions.
- **Outcome**: Incidence of MI.

Studies were included if they were as follows:

1. The association between RVO and risk of MI was also evaluated.
2. Provided sufficient data to calculate hazard ratios (HR) with 95% confidence intervals (CI).
3. Were peer-reviewed and published in English.

Studies were excluded if they:

1. Participants with pre-existing conditions that could confound the results, such as other severe cardiovascular diseases unrelated to RVO.
2. Were reviews, editorials, or case reports without original data.
3. Did not report necessary statistical measures for meta-analysis.

### Outcome measures and data extraction

The primary outcome of interest was the association between RVO and risk of MI. Data in the form of effect sizes, such as risk, hazard, and odds ratios, were pooled to assess this association. Further subgroup analyses were conducted to differentiate between central retinal vein occlusion (CRVO) and branch retinal vein occlusion (BRVO) in order to provide a more detailed understanding of the risks associated with each condition. Baseline data extracted from each study included author, year, country, sample size, age, sex (male), and presence of diabetes mellitus (DM). Two authors (K. Y. C. and H.C.C.) independently performed the data extraction process to reduce the likelihood of bias and mistakes. The extracted data was subsequently checked by a third author (C. M. C.) to ensure precision and coherence. Any discrepancies were addressed through discussion and agreement among the authors. This thorough approach guaranteed the reliability of the data used in the analysis and the sturdiness of the study’s findings.

### Quality assessment

We employed the Newcastle-Ottawa Quality Assessment Scale to assess the risk of bias [19]. The representation of the intervention cohort and selection of the non-intervention cohort were scrutinized for adequacy and representativeness. Correct intervention utilization was assessed to ensure methodological precision. Additionally, the relevance of the outcome of interest at the onset of the study was examined. Comparability between cohorts was evaluated based on age, sex, and injury severity, as well as other pertinent factors identified in the study design or analysis. The assessment also included whether the outcome was appropriately assessed and whether the follow-up duration was sufficient for the measured outcomes to manifest. Moreover, the adequacy of the cohort follow-up duration was considered to ascertain the reliability of the study’s findings. Through this comprehensive evaluation, the meta-analysis maintained a robust methodology, enhancing the credibility and validity of its conclusions.

### Statistical analysis

Statistical analysis was conducted using Comprehensive Meta-Analysis version 3.3. Hazard ratios with corresponding 95% confidence intervals were employed to pool the data, offering a robust measure of the effect size. A random-effects model was chosen for its ability to account for variability between studies and to enhance generalizability. Significance was determined by p-values less than 0.05, aligning with conventional standards. Heterogeneity was assessed using the Higgins I^2^ statistic, with values greater than 50% indicating significant heterogeneity among the included studies. Furthermore, publication bias was rigorously evaluated through visual inspection of funnel plots and supplemented by the Egger test to provide a quantitative measure.

## Results

### Study selection

**Fig. 1:**
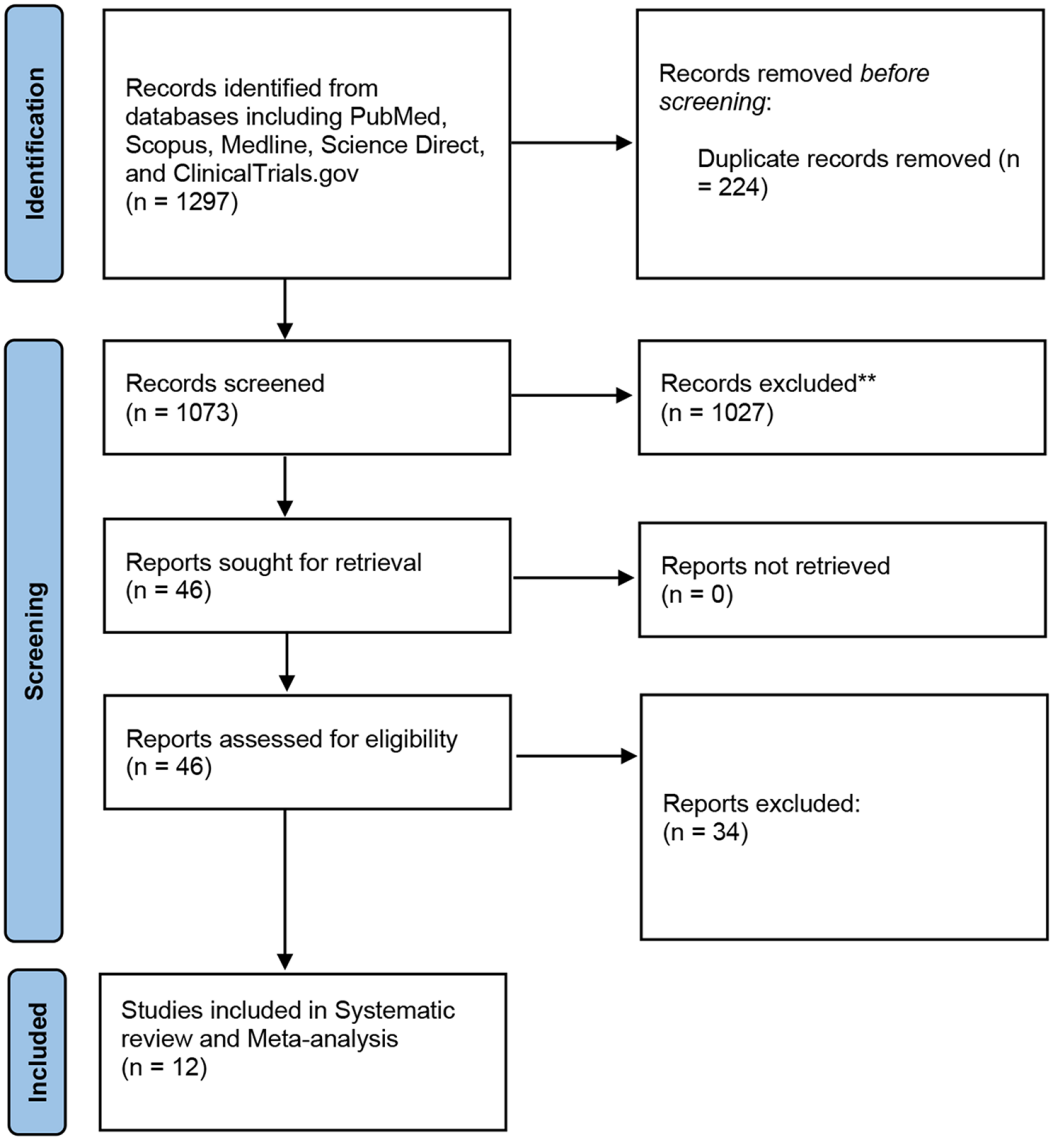
PRISMA flow diagram illustrating the study selection process for systematic review and meta-analysis. **Not meeting the eligibility Criteria

In the study selection process, a meticulous approach was employed to ensure the inclusion of relevant high-quality studies. A comprehensive search across multiple databases, including PubMed, Scopus, Medline, ScienceDirect, and ClinicalTrials.gov, initially yielded 1297 articles. After the removal of 224 duplicate records, 1073 unique articles underwent further evaluation. Screening of titles and abstracts resulted in the identification of 46 articles deemed potentially relevant for inclusion, thus warranting a thorough full-text review. Subsequently, following a stringent assessment against pre-established inclusion and exclusion criteria, 12 studies [20–31] met the necessary criteria for systematic review and meta-analysis (Fig. 1).

### Baseline characteristics

The baseline characteristics of the studies included in the meta-analysis are summarized in table below. The sample sizes varied widely, ranging from 45 participants in the study by Capua et al. [22](2012) to 45,304 participants in the study by Wai et al. [31](2024). The age of participants spanned a broad range, with most studies reporting mean ages in the 60s, except for Shih et al. [25](2015), which had a mean age of 79.6 years, and Umeya et al. [29](2021), which reported a mean age of 76.8 years. The proportion of male participants ranged from 40.4% in the study by Rim et al. [27](2016) to 68.8% in the study by Wai et al. [31](2024). The prevalence of comorbidities was also reported variably across studies, with DM prevalence ranging from 7% in Capua et al. (2012) to 49.7% in Rim et al. [27](2016), hypertension (HTN) prevalence ranging from 16.1% in Capua et al. (2012) to 60.9% in Hsieh et al. [26](2016), and hyperlipidemia prevalence ranging from 36.6% in Rim et al. [27](2016) to 76.4% in Hsieh et al. [26](2016). These studies were conducted in diverse countries, including Taiwan, the USA, Italy, Denmark, Korea, and Japan, reflecting a wide geographic distribution. The baseline characteristics of the included studies are shown in Table 1.

**Table 1:** Baseline characteristics of included studies

Study, authors (year); country	Study Design	Sample Size		Age (Years)		Gender (male)		Diabetes Mellitus (*n*)		Hypertension (*n*)		Hyperlipidaemia (*n*)			Conclusion
		Patients with RVO	Controls	Patients with RVO	Controls	Patients with RVO	Controls	Patients with RVO	Controls	Patients with RVO	Controls	Patients with RVO	Controls		
Hu et al. (2009) [20]; Taiwan	Retrospective Cohort	591	2955	50-59(131), 60-69(185), >70(190)	50-59(625), 60-69(925), >70(950)	297	1485	193	694	435	1449	219		733	RVO did not independently elevate the risk of acute myocardial infarction.
Werther et al. (2011) [21]; USA	Retrospective Cohort	4500	13,500	64.0 (13.4)	64.0 (13.3)	2239	6717	807	1493	2125	4215	1303		3210	The incidence of MI was comparable between patients with RVO and control groups; however, the rate of CVA in RVO patients was nearly twice that of the controls.
Capua et al. (2012) [22]; Italy	Retrospective Cohort	45	145	54.1 (14)	53.8 (13.5)	26	77	5	7	24	24	26		64	Coronary artery disease and non-fatal ischemic stroke were more prevalent in individuals with a history of RVO compared to a large cohort matched for cardiovascular risk factors.
Bertelsen et al. (2012) [23]; Denmark	Prospective Case-control	1168	116,800	50-59(235), 60-69(378), 70-79(330)	50-59(31383), 60-69(24 627), 70-79(16 323)	549	55,793	42	1547	188	14,688	NR		NR	Diabetes, hypertension, and peripheral artery disease increase the risk of developing branch retinal vein occlusion up to a decade later. Branch retinal vein occlusion, in turn, raises the risk of hypertension, diabetes, congestive heart failure, and cerebrovascular disease, highlighting the importance of preventive measures.
Bertelsen et al. (2014) [24]; Denmark	Retrospective Cohort	439	2195	50-59(60), 60-69(111), 70-79(139)	50-59(304), 60-69(554), 70-79(679)	230	1150	42	113	178	719	NR		NR	CRVO increased overall mortality compared to controls due to statistically attributed cardiovascular and diabetic disorders.
Shih et al. (2015) [25]; Taiwan	Retrospective Cohort	10,081	40,324	79.6 (4.8)	79.6 (4.8)	5578	22,312	3856	13,082	8197	28,363	3571		12,013	The findings of this research indicate a reciprocal relationship between the likelihood of developing comorbidities and the incidence of RVO in older individuals.
Hsieh et al. (2016) [26]; Taiwan	Retrospective Cohort	463	2315	40-50(100), 51-50(133), 61-80(230)	40-50(508), 51-60(651), 61-80(1156)	218	1,090	112	618	357	1326	110		407	The risk of mortality and atherosclerotic events was higher in patients who underwent incident haemodialysis and subsequently experienced retinal vascular occlusion.
Rim et al. (2016) [27]; Korea	Retrospective Cohort	1677	8637	50-59(393), 60-69(616), 70-79(334)	50-59(1963), 60-69(3076), 70-79(1662)	733	3659	1093	4383	282	2796	443		3205	RVO was connected with the formation of AMI, once adjustments were made for any possible confounding factors.
Chen et al. (2017) [28]; Taiwan	Retrospective Cohort	37,921	113,763	62.4±13.1	62.4±13.2	19,416	58,249	15,020	35,264	30,194	69,547	18,852		45,142	Individuals with RVO are at a significantly higher risk of developing AMI compared to those without RVO.
Umeya et al. (2021) [29]; Japan	Retrospective Cohort	57	125	76.8 (9.4)	75.6 (8.6)	23	43	16	26	6	0	NR		NR	This study uncovered a greater likelihood of cardiovascular events in individuals with RVO
Wai et al. (2023) [30]; USA	Retrospective Cohort	34,874		66 (15.2)		17 146		9174		19,277		16,995			This research found that individuals with RAO, both in the short and long term, had a higher risk of death, stroke, and MI when compared to those with cataracts.
Wai et al. (2024) [31]; USA	Retrospective Cohort	45,304	1,207,416	68.1 (14.3)	65.1 (10.8)	21,112	509,529	12,776	359,810	26,377	579,559	14,180		404,484	The incidence of mortality, cerebrovascular accident, and myocardial infarction is greater in patients with RVO than in their corresponding controls.

### Association between RVO and MI risk

**Fig. 2:**
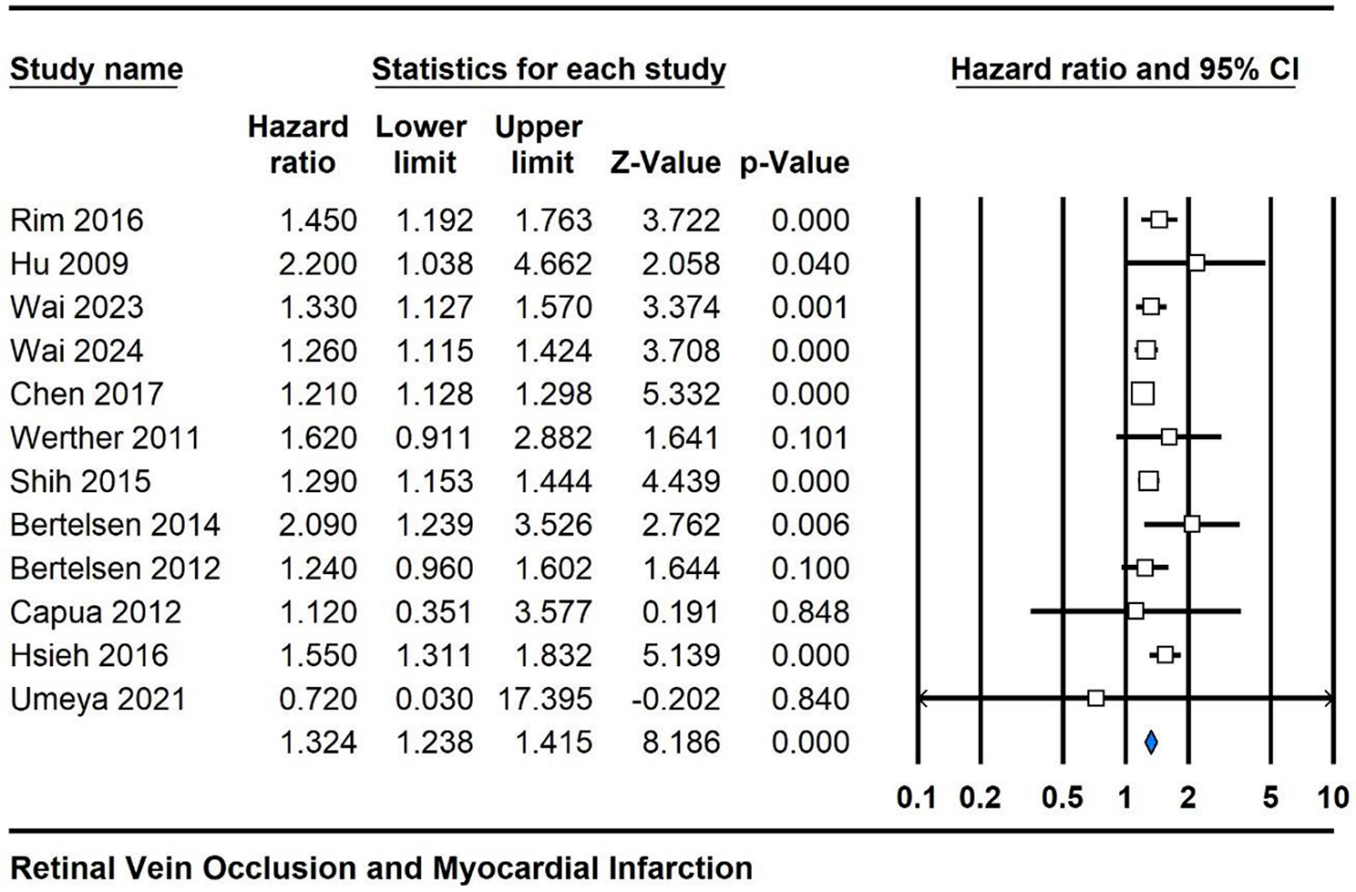
Forest plot depicting the pooled hazard ratio for the association between RVO and MI risk across twelve cohort studies

Across the 12 studies included in this meta-analysis evaluating the relationship between RVO and the risk of MI, the pooled hazard ratio was 1.324 (95% CI 1.238, 1.415), indicating a statistically significant positive association. The associated p-value was 0.0001, emphasizing the robustness of this association. Moderate heterogeneity was observed among the studies, with an I^2^ value of 29%, suggesting that a considerable portion of the variability in the effect estimates could be attributed to true differences between studies. These findings underscore the clinically relevant connection between RVO and elevated risk of MI, warranting further investigation and potential clinical implications (Fig. 2).

### Subgroup analysis

**Fig. 3:**
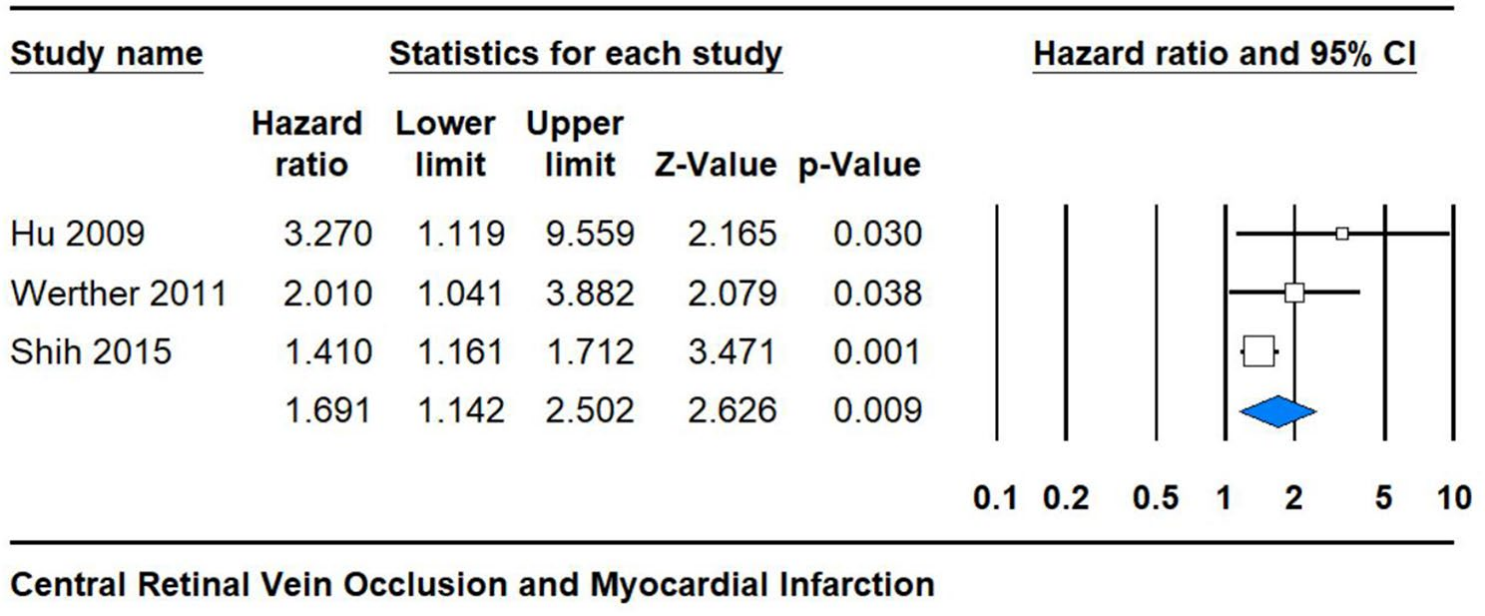
Subgroup analysis of CRVO showing the hazard ratio and confidence intervals for the association with MI risk

The analysis focused on CRVO revealed a statistically significant hazard ratio of 1.691 (95% CI 1.142, 2.502) with a low p-value of 0.009 and moderate heterogeneity (I^2^ = 36%). This finding suggests a robust association between CRVO and a heightened risk of MI. The narrow confidence interval further strengthens the precision of this estimate, underlining the reliability of the observed association. These results underscore the potential clinical significance of CRVO as a marker for an increased risk of cardiovascular events, highlighting the importance of early detection and management strategies to mitigate adverse outcomes in affected individuals (Fig. 3).

**Fig. 4:**
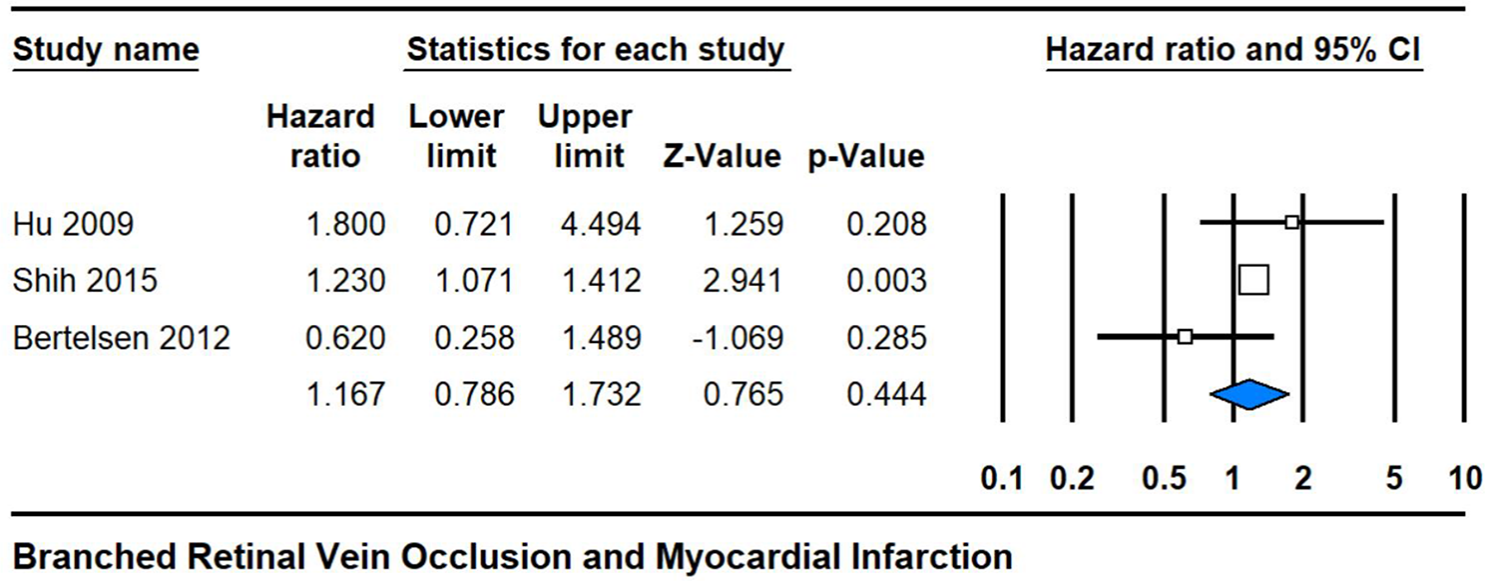
Subgroup analysis of BRVO showing the hazard ratio and confidence intervals for the association with MI risk

Conversely, the analysis pertaining to BRVO yielded a hazard ratio of 1.167 (95% CI 0.843, 2.106), with a non-significant p-value of 0.444 and moderate heterogeneity (I^2^ = 33%). While this estimate suggests a potential association between BRVO and MI risk, the wide confidence interval and non-significant p-value indicate uncertainty regarding the strength and significance of this relationship. The observed heterogeneity further suggests variability in the effect estimates across studies, potentially reflecting differences in the sample characteristics or methodological approaches. Thus, further research is warranted to elucidate the precise nature of the association between BRVO and cardiovascular risk, with implications for tailored clinical management strategies and risk assessments in affected populations (Fig. 4).

### Risk of bias and publication bias analysis

**Fig. 5:**
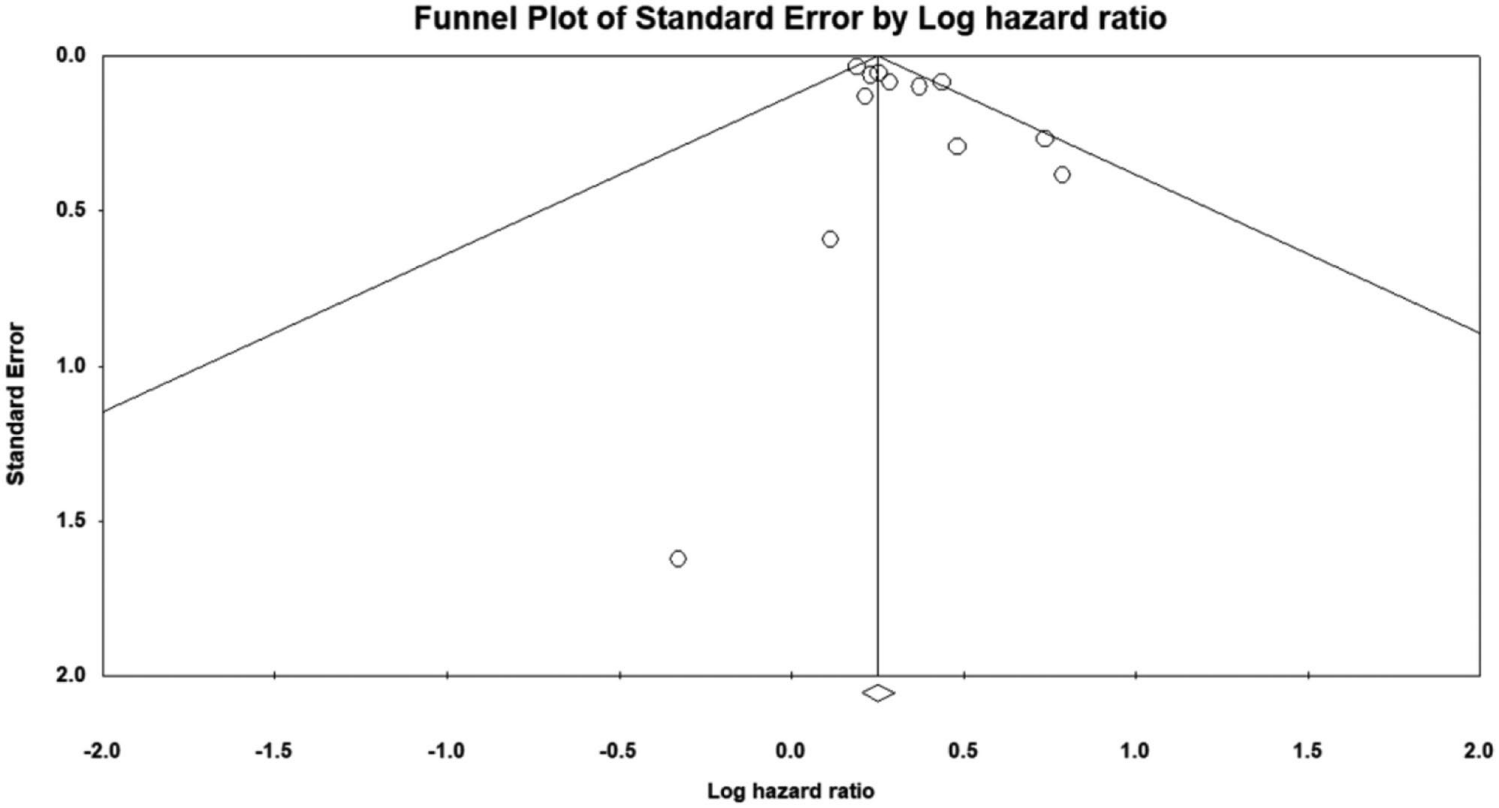
Funnel plot assessing publication bias in studies investigating the association between RVO and MI risk

A publication bias assessment was conducted to evaluate the meta-analysis’s robustness, revealing slight asymmetry in the funnel plot and potential publication bias. The Egger test indicated an intercept of 0.963 with a significant p-value of 0.036, confirming this bias. The Duval and Tweedie trim-and-fill test identified one study for trimming; post-adjustment, the hazard ratio was 1.32 (95% CI 1.25, 1.41) before and 1.32 (95% CI 1.24, 1.41) after adjustment. These results indicate that the association between RVO and MI risk remains significant despite publication bias, with slightly attenuated effect estimates (Fig. 5). The Newcastle-Ottawa Scale was used to assess bias risk in included studies, focusing on selection, comparability, and outcome domains. Most studies showed low selection bias, consistently scoring high on criteria such as cohort representativeness, non-intervention cohort selection, correct intervention use, and outcome presence at baseline. Studies by Hu et al. [20](2009), Capua et al. [22](2012), Bertelsen et al. [23, 24](2012, 2014), Shih et al. [25](2015), Hsieh et al. [26](2016), Rim et al. [27](2016), Chen et al. [28](2017), Umeya et al. [29](2021), and Wai et al. [31](2024) met al.l selection criteria. However, the comparability domain varied; most studies matched key factors like age, sex, and injury severity, but Werther et al. [21](2011) and Wai et al. [30](2023) did not meet the additional comparability criterion. In the outcome domain, all studies except Werther et al. [21](2011) met the criteria for outcome assessment and adequate follow-up duration. Overall, the studies displayed a low risk of bias, ensuring reliable and comparable meta-analysis results (Table 2).

**Table 2:** Summary of quality assessment for included studies using the Newcastle-Ottawa Scale for cohort studies, evaluating selection, comparability, and outcome domains

Newcastle Ottawa Quality Assessment Scale											
Study name			Selection				Comparability		Outcome		
			1	2	3	4	5	6	7	8	9
Hu et al. [20] (2009)			*	*	*	*	*	-	*	*	*
Werther et al. [21] (2011)			*	*	*	*	*	-	*	*	-
Capua et al. [22] (2012)			*	*	*	*	*	-	*	*	*
Bertelsen et al. [23] (2012)			*	*	*	*	*	*	*	*	*
Bertelsen et al. [24] (2014)			*	*	*	*	*	*	*	*	*
Shih et al. [25] (2015)			*	*	*	*	*	*	*	*	*
Hsieh et al. [26] (2016)			*	*	*	*	*	-	*	*	*
Rim et al. [27] (2016)			*	*	*	*	*	*	*	*	*
Chen et al. [28] (2017)			*	*	*	*	*	*	*	*	*
Umeya et al. [29] (2021)			*	*	*	*	*	-	*	*	*
Wai et al. [30] (2023)			*	-	*	*	*	-	*	*	*
Wai et al. [31] (2024)			*	*	*	*	*	*	*	*	*
Newcastle-Ottawa Quality Assessment Scale											
Selection:	1	Representation of the intervention cohort									
	2	Selection of the non-intervention cohort									
	3	Has the correct intervention been utilized?									
	4	Outcome of Interest present at the start of study?									
Comparability:	5	Are the cohorts comparable based on the design or analysis: age, sex, and injury severity?									
	6	Are the cohorts comparable based on the design or analysis? Additional factors									
Outcome:	7	Was the outcome assessed?									
	8	Was the follow-up long enough for measured outcomes to occur?									
	9	Was the cohort follow-up long enough?									

## Discussion

RVO and MI are vascular events that can significantly affect mortality. RVO occurs when the retinal blood supply is suddenly blocked, leading to acute vision loss, often indicating an underlying systemic vascular disease. MI, also known as a heart attack, occurs when blood flow to the heart is obstructed, causing damage to the heart muscle. Both conditions share common risk factors, such as hypertension, diabetes, and atherosclerosis, highlighting their significance in contributing to cardiovascular mortality. This meta-analysis aimed to investigate the association between RVO and the incidence of MI, providing a comprehensive synthesis of existing research. By identifying RVO as a potential marker of heightened MI risk, this study could facilitate early intervention and aggressive management of cardiovascular risk factors in affected individuals. By synthesizing data from multiple studies, we aimed to provide robust evidence that could influence clinical practice and emphasize the need for heightened cardiovascular monitoring in patients presenting with RVO. This study addresses a significant gap in the literature and seeks to enhance our understanding of the interconnected nature of ocular and cardiovascular health.

Our systematic review and meta-analysis assessed 12 studies to determine the relationship between RVO and the risk of MI. The combined hazard ratio revealed a statistically significant positive association, highlighting a noteworthy connection between RVO and an increased risk of MI. However, these studies showed moderate variability in their effect estimates. Subgroup analysis revealed a strong association between CRVO and MI risk, with high statistical significance and minimal variability. In contrast, the analysis for branched RVO showed a potential but non-significant association with MI risk, characterized by a wide confidence interval and moderate variability, indicating uncertainty and variability. The funnel plot showed slight asymmetry, which was confirmed by a significant Egger test. Adjustments using the Duval and Tweedie trim-and-fill test showed that while publication bias was present, the association between RVO and MI risk remained statistically significant, although slightly weaker after adjustment.

Multiple studies have indicated that individuals with RVO have a heightened risk of developing cardiovascular disease, particularly stroke [32–35]. These studies have emphasized that RVO often serves as an early warning sign for underlying systemic vascular diseases, which can manifest as stroke and other cardiovascular events. The identification of RVO as a predictor of stroke has led to recommendations for comprehensive systemic evaluation and prompt management of cardiovascular risk factors in patients presenting with RVO [36]. Our study expands this foundation by examining the association between RVO and MI, another critical cardiovascular event, thereby enhancing our understanding of the systemic implications of RVO. RVO develops when the retinal vein is obstructed and is typically caused by a disturbance in circulation or an embolus [37, 38]. The relationship between retinal vascular events and cardiovascular issues indicates common underlying comorbidities. Hypertension, hyperlipidemia, carotid artery disease, and diabetes mellitus are significant risk factors that contribute to both cardiovascular morbidity and mortality [38, 39]. Comprehensive population studies have demonstrated strong connections between systemic cardiovascular risk factors and RVO, emphasizing the critical roles of hypertension, elevated triglyceride levels, and renal dysfunction in these incidents [40, 41]. These findings underline the need for integrated management of cardiovascular and ocular health.

Studies have discovered that individuals with CRVO have a shorter life expectancy, living nearly a decade less than healthy individuals [42]. Another analysis revealed that mortality rates for patients with RVO were almost double those of patients without RVO over a protracted follow-up period [43]. In a study focusing on the European population, researchers found an all-cause mortality rate of 30% among participants over a 10-year follow-up; however, this study did not include a control group [44]. Another European study reported a considerably higher all-cause mortality rate within one year of RVO onset compared to the control group [45].

Age-related mechanisms of RVO could be responsible for the variations in the incidence of MI across different age groups. RVO might also serve as an additional risk factor for the development of MI, particularly in younger males [46]. Despite overall declines in MI morbidity and mortality, hospitalization rates for MI among young patients in the USA have not shown a decrease over the last ten years [47]. In contrast, in Korea, MI rates have risen in younger populations and declined in older individuals [16, 48]. Given these trends, it is essential to consider young patients with RVO as a high-risk group for cardiovascular disease, particularly in relation to MI [48]. MI is often caused by the natural progression of coronary atherosclerosis, with coronary artery occlusion resulting from plaques prone to rupture or erosion being the most common cause of MI [49]. Most studies conducted thus far have utilized MI mortality as their outcome measure, a method that might inadvertently downplay the incidence of MI [50, 51].

The development of RVO and MI involves a range of common mechanisms, including atherosclerosis, systemic inflammation, hypercoagulability, endothelial dysfunction, and embolism [36]. Atherosclerosis, which leads to the formation of plaques in the vascular walls, is a shared underlying factor for both conditions, resulting in vascular occlusion. Elevated levels of inflammatory markers are indicative of systemic inflammation, contributing to the progression of atherosclerosis and increasing the risk of both RVO and MI [49, 52]. Individuals with hypercoagulability, a state of heightened blood clotting potential, are more susceptible to thrombus formation in both the retinal and coronary vessels [53, 54]. Endothelial dysfunction caused by oxidative stress, hypertension, and diabetes impairs vascular homeostasis, further increasing the risk of blockage. Additionally, embolic phenomena, where clots or debris travel through the bloodstream and lodge in smaller vessels, can cause both RVO and MI, particularly in cases involving cardioembolic sources, such as atrial fibrillation [55]. These interconnected mechanisms underscore the systemic nature of vascular diseases and the importance of recognizing RVO as a potential marker of increased MI risk.

This meta-analysis exhibits several notable advantages. It is the first to provide a comprehensive and quantitative examination of the relationship between RVO and MI, addressing a significant gap in current research that previous analyses, which included studies with diverse control groups, did not cover [56–58]. By aggregating data from multiple sources, our study provides a more comprehensive understanding of the potential connection between RVO and MI compared to individual studies, thereby enhancing the reliability and clinical significance of the results. Furthermore, our analysis identifies crucial areas for future research, such as the necessity to control for confounding factors and establish uniform follow-up protocols. This thorough approach not only guides future studies but also contributes to the development of more precise guidelines for RVO patient management, ultimately enhancing patient care. Finally, this meta-analysis serves as a valuable tool for healthcare providers by increasing awareness of potential cardiovascular risks in RVO patients, potentially leading to more diligent monitoring and proactive cardiovascular management for improved patient outcomes.

This meta-analysis has various limitations that require acknowledgement. First, RVO patients are often referred to the emergency department upon acute presentation and subsequently not followed up in the ophthalmology department, leading to incomplete data on their long-term cardiovascular outcomes. Second, there is a scarcity of well-designed studies examining the link between RVO and MI, with many available studies having low quality, which may affect the reliability and generalizability of the findings. Furthermore, the meta-analysis is limited by the variability in the design and settings of the included studies, resulting in heterogeneity and complicating the synthesis of results. Another significant limitation is the lack of adjustment for confounders in several studies, with factors such as age and multiple comorbidities, which influence the prognosis and risk of MI, often inadequately accounted for, potentially affecting the results. Additionally, the follow-up periods in these studies varied widely, leading to inconsistencies in capturing long-term outcomes and true incidence of MI following RVO. These limitations underscore the need for more rigorous and well-designed studies with standardized methodologies and comprehensive follow-ups to better understand the relationship between RVO and MI.

## Conclusion

Our meta-analysis reveals a strong association between CRVO and a 69.1% increased risk of MI, while BRVO shows no significant correlation. When pooled together, RVO is linked to a 32.4% elevated risk of MI. Given the global prevalence of MI, our findings suggest a greater association between CRVO and MI compared to individuals without RVO. Despite slight publication bias, adjusted analyses confirm the reliability of these results. Our research indicates that improved cardiovascular monitoring for RVO patients, particularly those with CRVO, is essential to mitigate MI risk, underscoring the importance of proactive clinical care and further studies in this area.

## Electronic supplementary material

Below is the link to the electronic supplementary material.

Supplementary Material 1

Supplementary Material 2

## Data Availability

No datasets were generated or analysed during the current study.

## References

[1] Schorr EM, Rossi KC, Stein LK, Park BL, Tuhrim S, Dhamoon MS. Characteristics and Outcomes of Retinal Artery Occlusion: Nationally Representative Data, (in eng). Stroke. 2020;51(3):800–807.10.1161/strokeaha.119.027034. 10.1161/STROKEAHA.119.027034 31951154

[2] Hwang DD, et al. Incidence of retinal artery occlusion and related mortality in Korea, 2005 to 2018, (in eng). JAMA Netw Open. 2023;6(3):e233068. 10.1001/jamanetworkopen.2023.3068. 36897587 10.1001/jamanetworkopen.2023.3068 PMC12527418

[3] Liu W, Bai D, Kou L. Progress in central retinal artery occlusion: a narrative review, (in eng). J Int Med Res. 2023;51(9):3000605231198388. 10.1177/03000605231198388. 37712755 10.1177/03000605231198388 PMC10504844

[4] Monés J et al. Risk of Inflammation, Retinal Vasculitis, and Retinal Occlusion-Related Events with Brolucizumab: Post Hoc Review of HAWK and HARRIER, (in eng). Ophthalmology. 2021;128(7):1050–1059. 10.1016/j.ophtha.2020.11.011. 10.1016/j.ophtha.2020.11.011 33207259

[5] Talman V, Ruskoaho H. Cardiac fibrosis in myocardial infarction-from repair and remodeling to regeneration, (in eng). Cell Tissue Res. 2016;365(3):563 – 81. 10.1007/s00441-016-2431-9. 10.1007/s00441-016-2431-9 PMC5010608 27324127

[6] Chen R et al. Macrophages in cardiovascular diseases: molecular mechanisms and therapeutic targets, (in eng). Signal Transduct Target Ther. 2024;9(1):130. 10.1038/s41392-024-01840-1. 10.1038/s41392-024-01840-1 PMC11139930 38816371

[7] Cheung N et al. Traditional and novel cardiovascular risk factors for retinal vein occlusion: the multiethnic study of atherosclerosis, (in eng). Invest Ophthalmol Vis Sci. 2008;49(10):4297 – 302. 10.1167/iovs.08-1826. 10.1167/iovs.08-1826 PMC2584770 18539932

[8] Aslan Sirakaya H, Sirakaya E. Association of triglyceride–glucose index in branch retinal vein occlusion, (in eng). Graefes Arch Clin Exp Ophthalmol. 2024. 10.1007/s00417-024-06376-2. 38300335 10.1007/s00417-024-06376-2 PMC11222183

[9] Lee KE, et al. Management of Acute Central Retinal artery occlusion, a retinal stroke: an Institutional Series and Literature Review, (in eng). J Stroke Cerebrovasc Dis. 2021;30(2):105531. 10.1016/j.jstrokecerebrovasdis.2020.105531. 33310593 10.1016/j.jstrokecerebrovasdis.2020.105531

[10] Liu Y, et al. The impact of the Initial Admission Department on the management and prognosis of retinal artery occlusion, (in eng). Curr Neurovasc Res. 2022;19(5):440–8. 10.2174/1567202620666221027091249. 36305143 10.2174/1567202620666221027091249

[11] Woo SC, Lip GY, Lip PL. Associations of retinal artery occlusion and retinal vein occlusion to mortality, stroke, and myocardial infarction: a systematic review, (in eng). Eye (Lond). 2016;30(8):1031-8. 10.1038/eye.2016.111. 10.1038/eye.2016.111 PMC4985669 27256303

[12] Zhou Y, Zhu W, Wang C. Relationship between retinal vascular occlusions and incident cerebrovascular diseases: A systematic review and meta-analysis, (in eng). Medicine (Baltimore). 2016;95(26):e4075. 10.1097/md.0000000000004075. 10.1097/MD.0000000000004075 PMC4937964 27368050

[13] Roskal-Wałek J, et al. Long-term mortality after retinal artery occlusion - a single centre study, (in eng). Ann Agric Environ Med. 2023;30(2):252–8. 10.26444/aaem/167379. 37387374 10.26444/aaem/167379

[14] Salari N, et al. The global prevalence of myocardial infarction: a systematic review and meta-analysis, (in eng). BMC Cardiovasc Disord. 2023;23(1):206. 10.1186/s12872-023-03231-w. 37087452 10.1186/s12872-023-03231-w PMC10122825

[15] Weight N et al. Socioeconomic disparities in the management and outcomes of acute myocardial infarction, (in eng). Heart. 2023;110(2):122–131. 10.1136/heartjnl-2023-322601. 10.1136/heartjnl-2023-322601 37558395

[16] Kim RB, et al. Trends in the incidence of hospitalized acute myocardial infarction and stroke in Korea, 2006–2010, (in eng). J Korean Med Sci. 2013;28(1):16–24. 10.3346/jkms.2013.28.1.16. 23341707 10.3346/jkms.2013.28.1.16 PMC3546096

[17] Page MJ et al. The PRISMA 2020 statement: an updated guideline for reporting systematic reviews, (in eng). Bmj. 2021;372:n71. 10.1136/bmj.n71. 10.1136/bmj.n71 PMC8005924 33782057

[18] Brooke BS, Schwartz TA, Pawlik TM. MOOSE Reporting guidelines for Meta-analyses of Observational studies, (in eng). JAMA Surg. 2021;156(8):787–8. 10.1001/jamasurg.2021.0522. 33825847 10.1001/jamasurg.2021.0522

[19] Stang A. Critical evaluation of the Newcastle-Ottawa scale for the assessment of the quality of nonrandomized studies in meta-analyses, (in eng). Eur J Epidemiol. 2010;25(9):603–5. 10.1007/s10654-010-9491-z. 20652370 10.1007/s10654-010-9491-z

[20] Hu CC, Ho JD, Lin HC. Retinal vein occlusion and the risk of acute myocardial infarction (correction of infraction): a 3-year follow-up study, (in eng). Br J Ophthalmol. 2009;93(6):717 – 20. 10.1136/bjo.2008.151605. 10.1136/bjo.2008.151605 19208680

[21] Werther W, Chu L, Holekamp N, Do DV, Rubio RG. Myocardial infarction and cerebrovascular accident in patients with retinal vein occlusion, (in eng). Arch Ophthalmol. 2011;129(3):326 – 31. 10.1001/archophthalmol.2011.2. 10.1001/archophthalmol.2011.2 21402990

[22] Capua MD et al. Coronary artery disease, cerebral non-fatal ischemic stroke in retinal vein occlusion: an 8-yr follow-up, (in eng). Nutr Metab Cardiovasc Dis. 2012;22(1):23 – 7. 10.1016/j.numecd.2010.03.008. 10.1016/j.numecd.2010.03.008 20674314

[23] Bertelsen M, et al. Comorbidity in patients with branch retinal vein occlusion: case-control study (in eng). BMJ. 2012;345:e7885. 10.1136/bmj.e7885. 23204001 10.1136/bmj.e7885 PMC3510781

[24] Bertelsen M, Linneberg A, Christoffersen N, Vorum H, Gade E, Larsen M. Mortality in patients with central retinal vein occlusion, (in eng). Ophthalmology. 2014;121(3):637 – 42. 10.1016/j.ophtha.2013.07.025. 10.1016/j.ophtha.2013.07.025 24053999

[25] Shih CH, Ou SY, Shih CJ, Chen YT, Ou SM, Lee YJ. Bidirectional association between the risk of comorbidities and the diagnosis of retinal vein occlusion in an elderly population: a nationwide population-based study, (in eng). Int J Cardiol. 2015;178:256–61. 10.1016/j.ijcard.2014.10.110. 25464265 10.1016/j.ijcard.2014.10.110

[26] Hsieh TC et al. Risk of Mortality and of Atherosclerotic Events Among Patients Who Underwent Hemodialysis and Subsequently Developed Retinal Vascular Occlusion: A Taiwanese Retrospective Cohort Study, (in eng). JAMA Ophthalmol. 2016;134(2):196–203. 10.1001/jamaophthalmol.2015.5052. 10.1001/jamaophthalmol.2015.5052 26720586

[27] Rim TH, Han JS, Oh J, Kim DW, Kang SM, Chung EJ. Retinal vein occlusion and the risk of acute myocardial infarction development: a 12-year nationwide cohort study, (in eng). Sci Rep. 2016;6(22351). 10.1038/srep22351. 10.1038/srep22351 PMC4770309 26924150

[28] Chen YY, Sheu SJ, Hu HY, Chu D, Chou P. Association between retinal vein occlusion and an increased risk of acute myocardial infarction: a nationwide population-based follow-up study, (in eng). PLoS ONE. 2017;12(9):e0184016. 10.1371/journal.pone.0184016. 28898259 10.1371/journal.pone.0184016 PMC5595302

[29] Umeya R, Yoshida Y, Ono K. Impact of retinal vein occlusion on cardiovascular events in elderly Japanese patients, (in eng). Medicine (Baltimore). 2021;100(52):e28424. 10.1097/md.0000000000028424. 10.1097/MD.0000000000028424 PMC8718221 34967379

[30] Wai KM, et al. Risk of stroke, myocardial infarction, and death after retinal artery occlusion, (in eng). JAMA Ophthalmol. 2023;141(12):1110–6. 10.1001/jamaophthalmol.2023.4716. 37883068 10.1001/jamaophthalmol.2023.4716 PMC10603578

[31] Wai KM, Ludwig CA, Koo E, Parikh R, Mruthyunjaya P, Rahimy E. Risk of stroke, myocardial infarction, deep vein thrombosis, Pulmonary Embolism, and death after retinal vein occlusion, (in eng). Am J Ophthalmol. 2024;257:129–36. 10.1016/j.ajo.2023.08.022. 37660963 10.1016/j.ajo.2023.08.022 PMC13318398

[32] Bakhoum CY, et al. Retinal vein occlusion is associated with stroke independent of underlying cardiovascular disease. (in Eng) Eye (Lond). 2023;37(4):764–7. 10.1038/s41433-022-02038-x. 10.1038/s41433-022-02038-x PMC9998396 35411111

[33] Ørskov M, Vorum H, Larsen TB, Lip GYH, Bek T, Skjøth F. Similarities and differences in systemic risk factors for retinal artery occlusion and stroke: a Nationwide Case-Control Study, (in eng). J Stroke Cerebrovasc Dis. 2022;31(8):106610. 10.1016/j.jstrokecerebrovasdis.2022.106610. 35777081 10.1016/j.jstrokecerebrovasdis.2022.106610

[34] Subah G et al. Nationwide Incidence and Trends in Central Retinal Arterial Occlusion Management: A 5000-Patient Analysis, (in eng). Cardiol Rev. 2024;32(4):291–296. 10.1097/crd.0000000000000682. 10.1097/CRD.0000000000000682 38666795

[35] Kalva P, Akram R, Zuberi HZ, Kooner KS, United States. Prevalence and risk factors of retinal vein occlusion in the : The National Health and Nutrition Examination Survey, 2005 to 2008, (in eng). Proc (Bayl Univ Med Cent). 2023;36(3):335–340. 10.1080/08998280.2023.2173938. 10.1080/08998280.2023.2173938 PMC10120443 37091777

[36] Roskal-Wałek J, et al. Central and Branch Retinal artery Occlusion-Do they Harbor the same risk of further ischemic events? (in eng). J Clin Med. 2021;10(14). 10.3390/jcm10143093. 10.3390/jcm10143093 PMC8307136 34300257

[37] Chen TY, Uppuluri A, Zarbin MA, Bhagat N. Risk factors for central retinal vein occlusion in young adults, (in eng). Eur J Ophthalmol. 2021;31(5):2546–55. 10.1177/1120672120960333. 33008264 10.1177/1120672120960333

[38] Tauqeer Z, Bracha P, McGeehan B, VanderBeek BL. Hypercoagulability Testing and Hypercoagulable Disorders in Young Central Retinal Vein Occlusion Patients, (in eng). Ophthalmol Retina. 2022;6(1):37–42. 10.1016/j.oret.2021.03.009. 10.1016/j.oret.2021.03.009 PMC8460678 33774219

[39] Terao R, Fujino R, Ahmed T. Risk factors and treatment strategy for retinal vascular occlusive diseases, (in eng). J Clin Med. 2022;11(21). 10.3390/jcm11216340. 10.3390/jcm11216340 PMC9656338 36362567

[40] Guirado-Torrecillas L, Salazar-Rosa V. Retinal vein occlusion, a great unknown and a challenge in venous thromboembolic disease, (in eng). Rev Clin Esp (Barc). 2023;223(2):96–7. 10.1016/j.rceng.2023.01.001. 36669742 10.1016/j.rceng.2023.01.001

[41] Raviselvan M, Preethi B, Ratra D. Retinal perfusion density can predict cardiovascular disease risk in patients with retinal vein occlusion, (in eng). Indian J Ophthalmol. 2023;71(2):379–84. 10.4103/ijo.IJO_1662_22. 36727323 10.4103/ijo.IJO_1662_22 PMC10228965

[42] Su CK, Au SCL. Isolated and combined unilateral central retinal artery and vein occlusions after vaccination. A review of the literature, (in eng). J Stroke Cerebrovasc Dis. 2022;31(8):106552. 10.1016/j.jstrokecerebrovasdis.2022.106552. 35569402 10.1016/j.jstrokecerebrovasdis.2022.106552

[43] Wang JJ et al. Retinal arteriolar emboli and long-term mortality: pooled data analysis from two older populations, (in eng). Stroke. 2006;37(7):1833-6. 10.1161/01.Str.0000226929.23297.75. 10.1161/01.STR.0000226929.23297.75 16741179

[44] Vestergaard N, Torp-Pedersen C, Vorum H, Aasbjerg K. Risk of stroke, myocardial infarction, and death among patients with retinal artery occlusion and the Effect of Antithrombotic Treatment, (in eng). Transl Vis Sci Technol. 2021;10(11). 10.1167/tvst.10.11.2. 10.1167/tvst.10.11.2 PMC8419877 34468694

[45] Hankey GJ, Slattery JM, Warlow CP. Prognosis and prognostic factors of retinal infarction: a prospective cohort study, (in eng). Bmj. 1991;302(6775):499–504. 10.1136/bmj.302.6775.499. 10.1136/bmj.302.6775.499 PMC1669576 2012845

[46] Fong AC et al. Central retinal vein occlusion in young adults (papillophlebitis), (in eng). Retina. 1992;12(1):3–11. 10.1097/00006982-199212010-00002. 10.1097/00006982-199212010-00002 1565869

[47] Wong TY et al. Cardiovascular risk factors for retinal vein occlusion and arteriolar emboli: the Atherosclerosis Risk in Communities & Cardiovascular Health studies, (in eng). Ophthalmology. 2005;112(4):540-7. 10.1016/j.ophtha.2004.10.039. 10.1016/j.ophtha.2004.10.039 15808241

[48] Libby P. Current concepts of the pathogenesis of the acute coronary syndromes, (in eng). Circulation. 2001;104(3):365 – 72. 10.1161/01.cir.104.3.365. 10.1161/01.cir.104.3.365 11457759

[49] Marston NA et al. Association of Apolipoprotein B-Containing Lipoproteins and Risk of Myocardial Infarction in Individuals With and Without Atherosclerosis: Distinguishing Between Particle Concentration, Type, and Content, (in eng). JAMA Cardiol. 2022;7(3):250–256. 10.1001/jamacardio.2021.5083. 10.1001/jamacardio.2021.5083 PMC8590731 34773460

[50] Cugati S et al. Retinal vein occlusion and vascular mortality: pooled data analysis of 2 population-based cohorts, (in eng). Ophthalmology. 2007;114(3):520-4. 10.1016/j.ophtha.2006.06.061. 10.1016/j.ophtha.2006.06.061 17141315

[51] Tsaloumas MD, et al. Nine year follow-up study of morbidity and mortality in retinal vein occlusion, (in eng). Eye (Lond). 2000;14:821–7. 10.1038/eye.2000.230. 11584836 10.1038/eye.2000.230

[52] Glavinovic T, Thanassoulis G, de Graaf J, Couture P, Hegele RA, Sniderman AD. Physiological bases for the superiority of apolipoprotein B over low-density lipoprotein cholesterol and Non-high-density Lipoprotein Cholesterol as a marker of Cardiovascular Risk, (in eng). J Am Heart Assoc. 2022;11(20):e025858. 10.1161/jaha.122.025858. 36216435 10.1161/JAHA.122.025858 PMC9673669

[53] Marcinkowska A, Cisiecki S, Rozalski M. Platelet and thrombophilia-related risk factors of retinal vein occlusion, (in eng). J Clin Med. 2021;10(14). 10.3390/jcm10143080. 10.3390/jcm10143080 PMC8306401 34300244

[54] Cellai AP et al. A hypercoagulable and hypofibrinolytic state is detectable by global methods in patients with retinal vein occlusion, (in eng). Atherosclerosis. 2012;224(1):97–101. 10.1016/j.atherosclerosis.2012.06.053. 10.1016/j.atherosclerosis.2012.06.053 22800650

[55] González Bores P, Napal Lecumberri JJ, de la Torre Hernández JM, González-Mesones B, Galán. Hernández Hernández, Nonvalvular atrial fibrillation and retinal vein occlusion: the Valdecilla Cohort, (in eng). Rev Clin Esp (Barc). 2023;223(2):77–83. 10.1016/j.rceng.2022.11.005. 36669741 10.1016/j.rceng.2022.11.005

[56] Wu CY, Riangwiwat T, Limpruttidham N, Rattanawong P, Rosen RB, Deobhakta A, Association of retinal vein occlusion with cardiovascular events and mortality: A systematic review and Meta-analysis, (in eng). Retina. 2019;39(9):1635–45. 10.1097/iae.0000000000002472. 10.1097/IAE.0000000000002472 30829987

[57] Zhong C, You S, Zhong X, Chen GC, Xu T, Zhang Y. Retinal vein occlusion and risk of cerebrovascular disease and myocardial infarction: a meta-analysis of cohort studies (in eng). Atherosclerosis. 2016;247:170–6. 10.1016/j.atherosclerosis.2016.02.024. 26922716 10.1016/j.atherosclerosis.2016.02.024

[58] Zhang W, Guo X, Jiang X, Liu J, Han X, Guo C. Retinal microvascular changes and risk of coronary heart disease: A systematic review and Meta-analysis (in eng). Retina. 2024;44(2):333–44. 10.1097/iae.0000000000003959. 10.1097/IAE.0000000000003959 37831943

