# Supplementary material for: Is retinal vein occlusion highly associated with an increased risk of myocardial infarction? A systematic review and meta-analysis
Source: Int J Retina Vitreous. 2024 Nov 12;10:86. doi: 10.1186/s40942-024-00606-9 (PMC11556175; doi:10.1186/s40942-024-00606-9)
Supplement: Supplementary file 2 — Supplementary Material 2 [file 40942_2024_606_MOESM2_ESM.docx]

**Search Strategy**

| **Database** | **Search Strategy** |
| --- | --- |
| PubMed/Medline | (("retinal vein occlusion"[MeSH Terms] OR ("retinal"[All Fields] AND "vein"[All Fields] AND "occlusion"[All Fields]) OR "retinal vein occlusion"[All Fields] OR "RVO"[All Fields] OR ("retinal vein occlusion"[MeSH Terms] OR ("retinal"[All Fields] AND "vein"[All Fields] AND "occlusion"[All Fields]) OR "retinal vein occlusion"[All Fields] OR ("branch"[All Fields] AND "retinal"[All Fields] AND "vein"[All Fields] AND "occlusion"[All Fields]) OR "branch retinal vein occlusion"[All Fields]) OR ("retinal vein occlusion"[MeSH Terms] OR ("retinal"[All Fields] AND "vein"[All Fields] AND "occlusion"[All Fields]) OR "retinal vein occlusion"[All Fields] OR ("central"[All Fields] AND "retinal"[All Fields] AND "vein"[All Fields] AND "occlusion"[All Fields]) OR "central retinal vein occlusion"[All Fields]) OR "CRVO"[All Fields] OR "BRVO"[All Fields]) AND ("myocardial infarction"[MeSH Terms] OR ("myocardial"[All Fields] AND "infarction"[All Fields]) OR "myocardial infarction"[All Fields])) OR ("microbiology"[MeSH Subheading] OR "microbiology"[All Fields] OR "mi"[All Fields]) OR (("acute"[All Fields] OR "acutely"[All Fields] OR "acutes"[All Fields]) AND ("myocardial infarction"[MeSH Terms] OR ("myocardial"[All Fields] AND "infarction"[All Fields]) OR "myocardial infarction"[All Fields])) OR ("acute coronary syndrome"[MeSH Terms] OR ("acute"[All Fields] AND "coronary"[All Fields] AND "syndrome"[All Fields]) OR "acute coronary syndrome"[All Fields]) OR (("acute"[All Fields] OR "acutely"[All Fields] OR "acutes"[All Fields]) AND ("cardiovascular diseases"[MeSH Terms] OR ("cardiovascular"[All Fields] AND "diseases"[All Fields]) OR "cardiovascular diseases"[All Fields] OR ("cardiac"[All Fields] AND "event"[All Fields]) OR "cardiac event"[All Fields])) |
| Scopus | ( TITLE-ABS-KEY ( retinal AND vein AND occlusion ) OR TITLE-ABS-KEY ( rvo ) OR TITLE-ABS-KEY ( branch AND retinal AND vein AND occlusion ) OR TITLE-ABS-KEY ( central AND retinal AND vein AND occlusion ) OR TITLE-ABS-KEY ( brvo ) OR TITLE-ABS-KEY ( crvo ) AND TITLE-ABS-KEY ( myocardial AND infarction ) OR TITLE-ABS-KEY ( mi ) OR TITLE-ABS-KEY ( acute AND myocardial AND infarction ) OR TITLE-ABS-KEY ( acute AND coronary AND syndrome ) OR TITLE-ABS-KEY ( heart AND attack ) OR TITLE-ABS-KEY ( acute AND cardiac AND event ) ) |
| ScienceDirect | (TITLE-ABSTR-KEY ("retinal vein occlusion") OR TITLE-ABSTR-KEY("RVO") OR TITLE-ABSTR-KEY ("branch retinal vein occlusion") OR TITLE-ABSTR-KEY ("central retinal vein occlusion") OR TITLE-ABSTR-KEY("BRVO") OR TITLE-ABSTR-KEY("CRVO"))  AND  (TITLE-ABSTR-KEY ("myocardial infarction") OR TITLE-ABSTR-KEY("MI") OR TITLE-ABSTR-KEY ("acute myocardial infarction") OR TITLE-ABSTR-KEY ("acute coronary syndrome") OR TITLE-ABSTR-KEY ("heart attack") OR TITLE-ABSTR-KEY ("acute cardiac event")) |
